# Collaborative Challenges of Multi-Cohort Projects in Pharmacogenetics—Why Time Is Essential for Meaningful Collaborations

**DOI:** 10.2196/36759

**Published:** 2022-09-29

**Authors:** Filippo Franchini, Katharina Kusejko, Catia Marzolini, Christoph Tellenbach, Simona Rossi, Susanne Stampf, Michael Koller, Jivko Stoyanov, Burkhard Möller, Alexander Benedikt Leichtle

**Affiliations:** 1 University Institute of Clinical Chemistry Inselspital - Bern University Hospital University of Bern Bern Switzerland; 2 Division of Infectious Diseases and Hospital Epidemiology University Hospital Zurich Zürich Switzerland; 3 Division of Infectious Diseases and Hospital Epidemiology University Hospital Basel University of Basel Basel Switzerland; 4 Swiss Clinical Quality Management in Rheumatic Diseases Zürich Switzerland; 5 Swiss Transplant Cohort Study Clinic for Transplantation Immunology and Nephrology University Hospital Basel Basel Switzerland; 6 Swiss Paraplegic Research Nottwil Switzerland; 7 Institute of Social and Preventive Medicine University of Bern Bern Switzerland; 8 University Clinics of Rheumatology and Immunology Inselspital - Bern University Hospital and University of Bern Bern Switzerland; 9 Center for Artificial Intelligence in Medicine University of Bern Bern Switzerland

**Keywords:** personalized medicine, guidelines, ethical, legal, and social implications, study, ethics, multicentric

## Abstract

Multi-cohort projects in medicine provide an opportunity to investigate scientific questions beyond the boundaries of a single institution and endeavor to increase the sample size for obtaining more reliable results. However, the complications of these kinds of collaborations arise during management, with many administrative hurdles. Hands-on approaches and lessons learned from previous collaborations provide solutions for optimized collaboration models. Here, we use our experience in running PGX-link, a Swiss multi-cohort project, to show the strategy we used to tackle different challenges from project setup to obtaining the relevant permits, including ethics approval. We set PGX-link in an international context because our struggles were similar to those encountered during the SYNCHROS (SYNergies for Cohorts in Health: integrating the ROle of all Stakeholders) project. We provide ad hoc solutions for cohorts, general project management strategies, and suggestions for unified protocols between cohorts that would ease current management hurdles. Project managers are not necessarily familiar with medical projects, and even if they are, they are not aware of the intricacies behind decision-making and consequently, of the time needed to set up multi-cohort collaborations. This paper is meant to be a brief overview of what we experienced with our multi-cohort project and provides the necessary practices for future managers.

## Background

Multi-cohort studies are increasingly important in medicine as they provide the opportunity to join data and join efforts to increase the sample size and investigate questions beyond the scope of a single institution. Multi-cohort projects not only encourage cross-boundary collaborations, but they also boost synergies between cohorts by providing staff and funding. There are multi-cohort projects dealing with complementary cohorts around a similar subject and projects dealing with generalist cohorts, but none of them are representative of the general population.

At the international level, there are many initiatives that aim to facilitate collaborations between cohorts. The SYNCHROS (SYNergies for Cohorts in Health: integrating the ROle of all Stakeholders) project, a 3-year project funded by the Horizon 2020 Program that started in 2019, successfully mapped 1000 multi-cohort projects in 11 countries with the intent to harmonize the coordination and data interoperability of multi-cohort projects in Europe and worldwide [1,2]. SYNCHROS has a focus on identifying the practical, methodological, ethical, and legal challenges that cohorts are facing and on ideas how to facilitate the development toward tapping in data, which can be valuable for personalized medicine. We also encountered similar challenges while setting up our multicentric and multi-cohort project.

In this paper, we focus on different cohorts dealing with intrinsically different patients who are complementary but form a heterogeneous multi-cohort environment. The project tries to answer a particular scientific question by using patients selected from that heterogeneous multi-cohort environment. Finding a common ground across multiple cohorts and a consensus to a particular multi-cohort scientific question can be difficult. Furthermore, different cohorts have specific purposes, focus areas, and policies as well as their own established methods of managing, collecting, and sharing data.

Here, we use the Swiss project, PGX-link, as an exemplary case study to show the complexity of setting up a 2-year multi-cohort project—in our case, in the field of pharmacogenetics. This project is a feasibility study, focused on building an infrastructure that connects clinical and pharmacogenomics data among 3 cohorts: the Swiss HIV Cohort Study (SHCS) [3], the Swiss Transplant Cohort Study (STCS) [4,5], and the Swiss Clinical Quality Management (SCQM) in rheumatic disease [6]. The 3 cohorts were chosen because they previously worked together. In the COVERALL (Corona VaccinE tRiAL pLatform) study, patients from STCS and SHCS were vaccinated for the purpose of a research project and sample shipment was coordinated between the cohorts [7]. The IDEAL (IDEntifier LinkAge between patient-centered research and heaLthcare providers) project, including STCS, SHCS, and SCQM, focuses on automatic ways of identifying patients with their cohort IDs within the hospital system [8]; this is crucial for improving the matching, and as a result, makes data extraction from the hospital systems more feasible. New applications for interoperable “National Data Streams” within the Swiss Personalized Health Network (SPHN) and Swiss National Science Foundation (SNSF) funding framework also aim to use a multi-cohort approach. Therefore, PGX-link is not the only effort toward more interoperability and improvement of data flow, and it benefited a lot from the fact that the 3 cohorts were not completely “strangers.” However, in the past, the 3 cohorts never joined forces for a study like PGX-link (a genetic study consisting of the development of an infrastructure to answer a scientific question) and, as a consequence, the process of obtaining the relevant approvals was never explored at a multi-cohort level.

The project infrastructure was funded by the SNSF within the context of the BioLink program fostering collaborations between cohorts and their biobanks. The laboratory work and genetic analyses were funded by the Bern Centre for Precision Medicine (BCPM). Because of their large size, multi-centric projects are often funded by different bodies. PGX-link is a good example of a multi-cohort project where the access to the 2 different fundings was bounded to different prerequisites that were not met at the same time. This added an additional layer of complexity.

We show the evolution of the study protocol from the initial SNSF grant proposal to the final submission to the cohorts’ scientific boards and ethics committees to receive data and samples. We highlight the obstacles that we encountered in setting up and managing PGX-link as a multi-cohort collaboration. We explain in detail the changes in the proposal necessary for obtaining the project approval by the ethics committee, and we provide suggestions and potential solutions that can improve and support such processes for future multi-cohort projects and likewise serve as a guidance for funding agencies. Although some of the examples that we will provide here are “Swiss-centric,” we believe that the underlying messages that such examples convey as well as the intracohort and intercohort dynamics that we describe can be useful also at an international level.

## The Fundamental Steps of the Project

**Figure 1:**
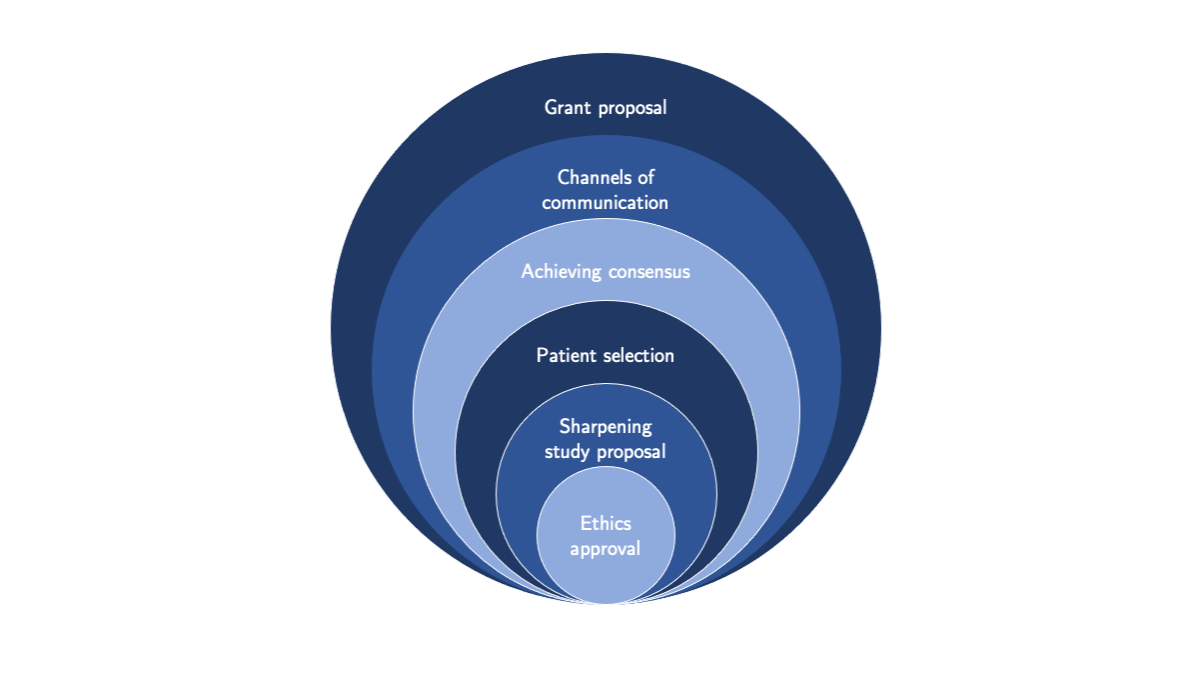
Consecutive spheres of collaborative endeavor of the PGX-link project from the grant proposal write-up through reaching intercohort consensus and the final ethics approval.

For PGX-link, we used an approach to answer a specific scientific question relying on a multi-cohort framework in a unique and ad hoc way. This strategy was preferred to using an infrastructure approach that would first set up an infrastructure and later on define the scientific questions that can be answered using the setup. Note that although from the cohort perspective, the scientific question approach adopts an ad hoc strategy, the question we want to answer acts as a common denominator across the cohorts joining the cohorts together. Thus, in the early stage of the PGX-link project, we went through the following 6 steps (see Figure 1):

1. Write-up of the grant proposal for the first funding body (SNSF) to fund the project infrastructure.
2. Establishing channels of communication with the cohorts.
3. Achieving consensus and feasibility discussion on a multi-cohort scientific question.
4. Patient selection within each cohort.
5. Sharpening of study proposal for the scientific boards.
6. Finalization of the study protocol and collection of the relevant documents to receive the approval from the ethics committee. The ethics committee approval is needed to retrieve funding from the second funding body (BCPM).

Although these steps might be slightly different or be in a different order in other countries, projects in medicine using software quality assurance will eventually require a cocktail of these steps to enable the retrieval of data and samples. Consensus on the scientific question is achieved after establishing the channel of communication with the cohorts. The scientific question is then used to select the patients, obtain approval from scientific boards and ethics committees, and access the BCPM funding.

At the end of each step, we critically reviewed our approach and gave suggestions to facilitate knowledge translation and successful collaboration. These steps formed a preparation phase that we used to set up all the “fundamental bricks” necessary to start the project. These bricks consist of compulsory paperwork to obtain the project approval from the cohorts and the ethics committees. As this long process includes sensitive data, it involves many people at different decision levels. Therefore, the preparation phase of a multi-cohort study takes a substantial amount of time (in our case, 1 year). This is a detail that is not obvious for funding bodies, which, in turn, are dependent on external assessment focusing on the scientific return on investment.

Cohort members and the ethics committee were extremely helpful and provided essential support to obtain the ethics approval necessary to start the project. Nevertheless, as this was a first-of-its-kind study in Switzerland, the bureaucratic process from step 1 to step 6 was not yet clearly perceivable. We thus decided to describe our journey and provide guidance for future multi-cohort studies. Note that, although the process described here might be transferable to other multi-cohort projects at national and international levels, some of the strategies that we used might not apply to other cohorts and regulatory circumstances.

## Step I: Writing the Grant Proposal

Before diving into the project evolution, it is important to know that each cohort has its own data/sample governance structure. The scientific board is responsible for evaluating the project proposal, especially the scientific question that the project aims to investigate. The foundation board is responsible for expressing the support to the project, and it includes legal representatives having the authority to sign documents on behalf of the cohort.

An ethics approval was necessary to receive the funding from BCPM and officially start the project (as such, funding was necessary to perform the genetic analysis). The ethics approval was conditional on approval from the scientific boards. In our case, p-v1 (proposal version 1, the first version of the proposal submitted to the SNSF) was approved by the foundation boards supporting data and sample sharing within our multi-cohort initiative, but it was not approved by the scientific boards. The SNSF BioLink grant specifically funded the infrastructure of the project. Still, a scientific question was required for successful submission, but it was not a key aspect of the grant. This scientific question was added to p-v1 close to its submission on the data available at this time and thus was not reviewed by the scientific boards. Hence, the evaluation of the scientific question by the scientific boards was independent of the support of the foundation boards.

The scientific boards were not involved because of organizational incompatibilities: it was not possible to receive feedback from the cohorts’ collaborators while writing the grant because deadlines were extremely short and during typical summer holiday times. Without in-depth knowledge of the cohort data and proper discussion between the cohorts themselves, the scientific question could only be superficially outlined. This explains why after receiving the SNSF grant, the scientific question in p-v1 was not entirely clear to the scientific boards. The scientific boards would only agree on p-v1 if the scientific question was clearly stated and was precise and feasible with enough patients available to guarantee the statistical power sufficient for genetic studies. Having enough time between grant call and deadlines to involve collaborators early on would have allowed the project management to be more efficient. Following the feedback of the scientific boards and the cohort collaborators, the proposal had to be thoroughly revised. We thus used a substantial amount of the project time and funding just for the proper preparation without even starting the actual project. To reach a consensus on a multi-cohort scientific question (ie, a feasible question that is of interest to all 3 cohorts), we set up the intercohort collaboration network. This led to discussions with cohort collaborators and scientific boards that helped to understand that the scientific question proposed in p-v1 was not sufficiently fitting the available data and too complicated for a genetics study within the given timeframe.

We experienced that not having the scientific question clearly defined from the beginning is detrimental to the project’s budget and timeline. Thus, for a multi-cohort project to be efficient, it is important to involve scientific board members and cohort collaborators early during the writing phase of the grant proposal—at least 1-2 people from each cohort who are experts in the field, who know “their” cohort’s data, and who can contribute at an early stage. Asking the funding bodies to have an early announced application process with an adequate duration of the presubmission phase is crucial for the proper allocation of people and resources. It is important to have sufficient time between the grant call and deadline to allow for appropriate communication between partners and for the scientific boards to adapt to the process. In this way, the proposal can benefit from feedback from the scientific boards.

## Step II: Setting Up Channels of Communication

In this step, we opened channels of communications between the cohorts, establishing the intercohort collaboration network, as well as with laboratory services, information technology services, and legal services to address all concerns from the cohorts and the ethics committee. For PGX-link, we had expert collaborators with different backgrounds on board: physicians, pharmacologists, and representatives of the cohorts involved in data management. This provided the project manager with strong scientific and practical support during discussions. The collaborators were approximately 10 people who were mostly active during the elaboration of the reviewed version of p-v1, that is, proposal v2.0 or p-v2. The implementation of the scientific boards’ feedback and the inclusion of detailed information collected from information technology services (data security and storage), laboratory services (costs and procedures to analyze the samples), and legal services (data and material transfer agreements) led to the official version of p-v2 ready to be submitted to the ethics committee.

An official review and a thorough discussion of the project are usually done during scientific board meetings that are conducted approximately 4 times a year. We would like to make the reader aware that cohort collaborators in the project are not necessarily part of the cohort scientific board committee. Involving only non–scientific board members would result in a proposal that does not address the concerns of the scientific board. This could lead to multiple review rounds, thereby increasing preparation time. In our case, we went through a minimum of 2 rounds in each cohort. Thus, to be time-efficient, we recommend that the project manager includes scientific board members at an early stage in the project so that (1) early concerns of the scientific board can be addressed and (2) project updates can be communicated beforehand to the other scientific board members. Multi-cohort projects depend on multiple scientific boards (one for each cohort) that within a year have a few meetings at different time points. Instead of meeting the scientific boards separately and dramatically increasing the preparation time, we encourage involving members of other cohorts’ scientific boards to the first available scientific board meeting. In this way, it is possible to obtain a more holistic view of the project and address multi-cohort concerns in 1 meeting. If not already standard practice, we also encourage the project management to participate in the scientific board meetings when the scientific board is discussing your project. This is the opportunity to present the project in person and answer upcoming questions. Besides these suggestions, we recommend standard best practices in project management such as (1) avoiding planning short notice meetings and using a widely accepted meeting planning software to plan meetings, (2) keeping an agenda of all the meetings, and (3) communicating the meeting agenda in advance and sharing the minutes shortly afterwards. Not following such points could result in collaborators who do not know who is attending the meeting, who do not find time to participate, who do not get an update where the project stands, and what the next steps are or what contribution is expected from them.

The sharpening of the scientific question was probably the most difficult task in the preparation phase of PGX-link, requiring all the cohorts to engage in discussion and find a consensus. Multi-cohort meetings created an occasion for joint discussions, making collaborators aware of what is collected in the other cohorts and adjust the question accordingly.

## Step III: Sharpening the Scientific Question

The number of prospective patients is necessary to demonstrate the feasibility of the study in terms of participant numbers needed for the statistical power and budget. Having the correct number of patients implies that the scientific question is clear and precise so that it is possible to define the inclusion and exclusion criteria for the patient selection. If there are not enough patients, the criteria need to be iteratively rediscussed and possibly adapted until a sufficient number is met (as the number of cohort participants cannot easily be expanded).

In the original grant proposal p-v1 and previously in the funding offer from the SNSF, the term “infrastructure” was kept relatively open, probably because in earlier stages, there was not enough knowledge about the general feasibility of such a study. Thus, as a feasibility study, instead of building an exhaustive infrastructure, we decided to devise a small cohort metadata set containing only the data necessary to answer the scientific question. The cohort metadata set can be explored using some raw code that can be viewed as core infrastructure (ie, a raw code reviewed by cohort collaborators that can be used to perform, eg, patient selection within a specific cohort). This can be scaled to include more data and allow a more user-friendly interaction. Such evolution was embraced by the cohorts as a more realistic approach than those initially proposed in p-v1.

With PGX-link, we focused on chronic kidney disease in patients mainly treated with antiviral or immunomodulatory medications that are known to bear a risk of renal impairment and investigated the genetic variants that are predictive of a decline in renal function. Some crucial technical and epidemiological assumptions/prerequirements had to be met: (1) common medication, (2) availability of creatinine measurements results and dates, (3) availability of biosamples, and (4) semantically interoperable data (or metadata). Points 1-3 revealed some real-world problems as patients within the different cohorts had different diseases. However, such scientific questioning presented important advantages. We used estimated glomerular filtration rate as the criterion to select patients for this study, as this value can be simply calculated using sex, age, and serum creatinine levels of the patients [9,10]. Creatinine levels are widely measured and accessible. Thus, we only needed a few basic data items that are routinely collected. Moreover, the existence of previous studies [11,12] focusing on renal impairment in patients within the same cohorts allowed us to (1) already have genetic data available and thus increase the number of patients, (2) have peer-reviewed methodology for patient selection by using estimated glomerular filtration rate values, and (3) have support from previous collaborators involved in patient selection and genetic analysis.

Thus, we recommend sticking with common best practices in project management. It is crucial that the right terminology and clear communication are used when we describe a project to cohorts. We also encourage to keep the study as simple as possible. Unexpected problems are always around the corner. This is important for any study and even more important for multi-cohort studies. Whenever possible, available methodologies or data should be used, as it will save time and resources. An additional hurdle is the fact that templates for letter of intent and full proposal could differ between the cohorts. If this is the case, it would be more efficient to have a standardized template for multi-cohort studies in order not to submit the same content multiple times in different formats.

## Step IV: Defining the Number of Participants

The number of participants is a crucial factor for the statistical power of a study and to obtain the approval from the scientific boards and the ethics committee. A clear scientific question and clear criteria are necessary for selecting the patients for a study. However, without the approval from the ethics committee (dependent on scientific board approval), it is not possible to obtain access to patients’ data. Therefore, how can we estimate the number of suitable patients if we do not have access to the data at the time of writing the proposal?

The approach we took was using remote patient selection in which the cohorts provide the structure of their database to the project manager who, in turn, devises a piece of software (in our case, an R-package on GitHub called PGX-link tools [13]) that the cohorts can use and run on their local secured infrastructure to provide a rough estimate of the number of potential patients. Although this step worked well, it required tight cooperation between the project manager and cohorts, since not having direct access to the data makes the development of the package more difficult. This procedure allows only broad estimates of the number of participants because the package might contain bugs that can be fixed only once the developer has access to the data, and additional exploratory analysis can reveal that some selected patients are nevertheless not eligible for the study. Still, in most cases, remote patient selection is precise enough to obtain the required approvals from the scientific boards and the ethics committee, with the caveat that numbers after final patient selection might differ. We suggest that the developer should access the data before submitting the proposal, as it increases precision in patient selection. Currently, the only way of doing that is by being physically present in the cohorts’ infrastructure and supported by a team member. The development of a tool to interact with cohorts’ data requires some time to devise. It is not only dependent on the developer but also on the cohorts’ collaborators, especially data managers and clinicians, which proved to be extremely supportive in our case. In the best case scenario, such a developmental stage requires probably 1-2 weeks and in the worst-case scenario, even up to 1-2 months. In our case, the cohort dealing with patients who underwent transplantation (STCS) presented a particular example since the time from transplantation to case definition (ie, follow-up period) added an additional layer of complexity. Another recently released option to perform remote patient selection could be a so-called cohort explorer [14], that is, a secured web-based infrastructure that can be used to explore data within a specific cohort. However, depending on the granularity of the queries, this might not always be sufficient.

Usually, cohort patients treated in Swiss hospitals have their data stored in the hospitals’ databases (it is even required by law that the hospitals keep the patient data records). Such data can include medication and routine measurements such as creatinine levels. In our case, the creatinine level data of 1 cohort were kept in 2 Swiss hospitals. To retrieve those creatinine values, additional paperwork was required to obtain the relevant permits. Be aware that there could be problems of underreporting: in our specific case, medication information retrieved from 1 hospital was not as detailed as that retrieved within the cohorts. It is thus important to double check with the cohorts any data that are retrieved externally, as the cohorts generally have more cautiously curated databases.

Regarding the analysis of biosamples, cohorts have their own policies. If a genetic database is already in place, it is very likely that they will prevent patchwork analyses from different projects but rather analyze many samples that will include most but not all samples needed for the project. Thus, it is important to have enough funding to participate in large genetic screenings that include samples from multiple projects, with the aim of getting your samples analyzed. Cohorts will always be happy to generate more genetic data for their patients to have a broader data foundation that can be potentially reused by other projects. However, from a project management point-of-view, the benefit of obtaining additional data from genetic analysis must outweigh the costs. If the cohorts already have genetic data available, it is good practice to use those data first, and then if strictly required, proceed with additional screening.

Thus, in this section, we recommend, whenever possible, to avoid remote patient selection and visit the cohorts to perform patient selection with actual patient data without working with mock data sets. We also encourage to use genetic data that are already available before proceeding with additional genetic analyses as they are expensive and are usually prone to substantial time delay especially if the cohorts are putting together multiple projects to screen a large number of patients.

## Steps V and VI: Obtaining Scientific Board and Ethics Committee Approvals

Two perspectives are intertwined in the progress of complex collaborations—reaching sound and consensual strategic decisions and timely decisions. The latter does not have to always wait for the former—the project timeline can benefit from a proactive anticipatory approach in which certain milestones are de facto prepared and ready to be used once a decision is reached on a strategic level. Many steps are strictly dependent on the previous ones, sometimes with insolvable circularities. Although these dependencies are in place to ensure security when dealing with patients’ sensitive data, scientific board and ethics committee approvals can be overlapping when being addressed jointly. The draft of p-v2 was already included in the ethics committee submission to gain time and to obtain the conditional ethics committee approval without waiting for the scientific board meetings. Thus, we obtained the conditional ethics committee approval before the proposal was officially accepted by all scientific boards (1 cohort officially accepted before submission to the ethics committee). With the conditional ethics committee approval, we were able to obtain the hospital approval to start the data search with the help of the data science services. Additionally, the conditional ethics committee approval probably increased the trust in the project by the cohorts.

Thus, we suggest not to wait for all the scientific boards to formally approve the final project plan. The application to the ethics committee should be started as soon as a viable version of the proposal has been approved by the collaborators and possibly contains some feedback from the cohort scientific boards. Ethics committee clearance is a formal and official process that usually takes some time. The approval will ease (but not speed up) the processes within the cohorts. Amendments to the protocols and ethics submissions might be necessary but are simpler *ex posteriori*.

## What to “Take Home”

In this paper, we illustrate how complex the preparation phase of a multi-cohort project in pharmacogenomics can be. We encountered multiple challenges along the way, and we believe they will likely affect other researchers conducting similar projects at national and international levels in the future as they have been already encountered in the past [1,2]. We expect that in the future, the number of funding opportunities for such projects will rise (compared to monocohort or monocentric projects) and these projects will provide opportunities to highlight current heterogeneities between different cohorts but likewise, unlock their great collaborative potentials. The SYNCHROS project was a €2 million research program that mapped 1000 multi-cohort projects funded by European, American, and Canadian institutions. Although the project is at its end, it is likely that such type of grants will continue in the future.

It is clear to us that heterogeneities between cohorts will always exist and remain because of the different nature of the cohort-specific diseases. However, there are some minor adaptations that can facilitate the future fluidity of multi-cohort studies. For example, each cohort has its own specific material and data transfer agreement, own proposal template, and own way of approaching data sharing and security. These challenges were previously mentioned at the international level, and the harmonization of such documents would ease collaboration at the national and international levels [15,16]. If all cohorts will share a common proposal template, material and data transfer agreement, and the system of sharing data, the processes will be simpler and seamless between the cohorts. Of course, this will require efforts not only from the cohorts but also from legal services involved in such types of legal contracts. We propose a unified template for the letter of intent and for a full proposal for all the national cohorts, while legal services could provide the material and data transfer agreement based on the centers involved in the project [17].

We noticed that despite national initiatives to improve synergies such as the Swiss Biobanking Platform (SBP) and SPHN, at the time of writing the proposal, the cohorts were still in the process of obtaining the newly released biobank labels of SBP or having their database adapted, as recently suggested by SPHN. Most of the cohorts are much longer established than SBP and SPHN and, as a consequence, such restructuring processes can interfere with the cohorts’ timelines, and more importantly, be very expensive. The current situation does not yet allow cohorts to invest time and resources in changing their system in favor of potential further collaborations, and money should be specifically allocated for this task. For example, the application process to obtain the quality label from SBP is detailed and requires help from laboratory members who are not paid by the cohort and most of the time help “in kind.” However, the immediate benefit of obtaining that label is that the cohort biobank has been checked and internal procedures such as protocols and type of laboratory equipment have been reviewed. Researchers that use biosamples from labeled biobanks can be sure that the samples they are using follow SBP standards, and this makes their methodology solid for potential publications. The management of biobanks at a multi-cohort level is essential to ease the process of patient selection or simply as an exploration tool for project management. This has been previously emphasized at the national and international levels to improve method harmonization and integration [16]. The extent of overlap and standardization between the cohorts will prospectively rise with specific funding for multi-cohort projects. Hence, funding agencies should incorporate the intricacies of this type of collaboration in their funding schemes.

Ideally, the cohorts increase synergies by starting regular multi-cohort communications, following the new standards of SBP and SPHN, and agreeing on common document templates such as letters of intent, project proposals, and data transfer agreements. Common variables (or metadata definitions) across the cohorts ensure a minimum viable meta-cohort data set that can be used in multi-cohort projects, especially in short-term feasibility studies. Such a data set can include routinely collected data that are common in all cohorts, such as gender, age, weight, height, medication (eg, via Anatomical Therapeutic Chemical codes), blood pressure, and standardized laboratory assessments. The feasibility of having a minimum viable data set needs discussion, as scientific questions are usually specific, and only with basic information, one cannot spark interesting projects. If these small steps are in place before a multi-cohort project starts, from the cohorts’ perspective, one would avoid patchwork from multiple projects requesting different data just to answer a particular question. In contrast, from the project management perspective, filling the same type of documents multiple times and performing multiple submissions of similar documents could be avoided. To make this happen, funding bodies must foster the use of common standards for all cohorts and funding must be available nationwide to ease the transition from a monocentric standpoint to a multi-cohort perspective. Furthermore, currently, little importance is given to the administrative, regulatory, and scientific setup of a multi-cohort that easily could take up to 1 year. The SYNCHROS project concludes that retrieving data, analyzing them, and publishing findings are not the only challenges in a multi-cohort project. A lot of funding and efforts must be devoted to address practical, methodological, ethical, and legal challenges that usually arise before the actual project starts, as there are existing infrastructures and governance variants for each involved cohort. Here, we suggest that it would be helpful if funding bodies could divide the grant that can be used to set up the project before it officially starts (ie, getting all the necessary approvals and clearance to sensitive data) from a grant to actually conduct the project. It is thus important that the scientific value of a project is clearly outlined and peer reviewed before proceeding with its execution. In our view, funding only the infrastructure is not enough, as an infrastructure must have a clear purpose and answer specific questions. We suggest that funding bodies should request approval from the scientific boards before releasing the grant. This will ensure that substantial additional preparatory work has been done by the grantees, as communication with cohorts is the key to get the scientific boards on board as we outlined in this paper.

## Abbreviations

BCPM: Bern Centre for Precision Medicine
SBP: Swiss Biobanking Platform
SCQM: Swiss Clinical Quality Management
SHCS: Swiss HIV Cohort Study
SNSF: Swiss National Science Foundation
SPHN: Swiss Personalized Health Network
STCS: Swiss Transplant Cohort Study
SYNCHROS: SYNergies for Cohorts in Health: integrating the ROle of all Stakeholders
